# Berberine improves inhibitory avoidance memory impairment of *Toxoplasma gondii*-infected rat model of ketamine-induced schizophrenia

**DOI:** 10.1186/s12906-023-04107-4

**Published:** 2023-08-30

**Authors:** Neghin Gholizadeh, Abdolhossein Dalimi, Fatemeh Ghaffarifar, Mehryar Nader-Mohammadi, Parviz Molavi, Masoomeh Dadkhah, Soheila Molaei

**Affiliations:** 1 Students Research Committee, Public Health School, Ardabil University of Medical Sciences, Ardabil, Iran; 2 Department of Parasitology, Faculty of Medical Sciences, Tarbiat Modares University, Tehran, Iran; 3 Department of Psychiatry, School of Medicine, Ardabil University of Medical Sciences, Ardabil, Iran; 4 Pharmaceutical Sciences Research Center, Ardabil University of Medical Sciences, Ardabil, Iran; 5 Zoonoses Research Center, Ardabil University of Medical Sciences, Ardabil, Iran

**Keywords:** Toxoplasma gondii, Schizophrenia rat model, Inhibitory avoidance memory, Consolidation, Reconsolidation, Berberine

## Abstract

**Background:**

Memory impairment caused by *Toxoplasma gondii* infection has been documented. *Berberine* (BRB) is well known for its enhancing effects on memory and has shown promising results. However, the impact of BRB on *T. gondii* infection and schizophrenia-induced consolidation and reconsolidation memory impairment is still unclear. Here; we examined the effect of BRB on the inhibitory avoidance (IA) memory consolidation and reconsolidation impairment induced by *T. gondii* infection, and ketamine (Ket) as a pharmacological model of schizophrenia. Also; the brain-derived neurotrophic factor (BDNF) levels in the medial prefrontal cortex (mPFC) and hippocampus were analyzed.

**Methods:**

Rats were infected with *T. gondii* RH strain or received Ket (30 mg/kg/day) intraperitoneally (i.p) for at least five consecutive days (as the model of schizophrenia). Then followed by oral administration with BRB (25 mg/kg/day) for five days. Finally, the IA memory retention test was examined 48 post-conditioning, and BDNF was measured.

**Results:**

Results indicated IA memory impairment in *T. gondii*-infected animals since lower step-through latency (STL) was observed than in control animals. We found significant (P = 0.01, P = 0.001) elevations in STL and a significant decrease (P = 0.001) in total time spent in the dark area following BRB administration in infected and Ket-treated rats, indicating improvement (increased STL) in consolidation and reconsolidation memory. Moreover, BDNF levels were reduced (P = 0.01) in the hippocampus and mPFC regions of both *T. gondii-* infected and Ket-induced groups, which remarkably enhanced after BRB treatment. Furthermore; we found that BRB administration notably increased the mPFC BDNF levels in mPFC (P < 0.01) and hippocampus (P = 0.001) in the Ket-treated and rats infected with *T. gondii*.

**Conclusion:**

Taken together; BRB may be a valuable preclinical treatment for improving memory impairment through BDNF expression in PFC and hippocampus, therefore; BRB is suggested for memory disturbances induced by *T. gondii* infection.

## Background

*T. gondii* is an obligate intracellular protozoan parasite that causes toxoplasmosis in a wide range of homeothermic hosts. Approximately one-third of the human population is infected by brain parasites, especially *T. gondii* [1]. Numerous studies have reported that *T. gondii* infection is one of the most risk factors for alterations in human behavior, personality, and mental disorders, such as schizophrenia [2, 3]. Schizophrenia is a chronic mental disorder that affects approximately 1% of the world’s population, making it the seventh most costly medical challenge [4]. It is distinguished by several symptoms including severe behavioral deficits and memory dysfunction [5]. On the other hand, schizophrenia results in impaired neurocognitive functioning, which includes processing speed, memory, learning, social cognition, and executive function [6]. In this regard, the infection can also cause memory impairment [7] and cognitive deficits [8]. Memory consolidation is a newly formed memory that needs gene expression for several hours to become stable or consolidated. In other words, after retrieval, the reactivated labile memory requires a gene expression-dependent process to be re-stabilized during memory reconsolidation which becomes stable again [9].

BDNF is one of the most mediators in regulating neurogenesis, differentiation, maturation, and neuronal survival during development [10]. Due to the high density of BDNF in the adult’s brain hippocampus and cerebral cortex, it could enhance synaptogenesis and neurotransmission [11], especially in several memory types of processing [12, 13].

Recently, the preventive and therapeutic effects of herbal medicines have been shown in some illnesses and infectious diseases [14]. BRB is an organic isoquinoline alkaloid compound isolated from different medicinal herbs such as *Berberis vulgaris* and has been widely used for medicinal therapeutic purposes [15]. Besides the anti-inflammatory and anti-apoptotic properties of BRB [16], it is capable to attenuate oxidative stress, improve ethanol-induced memory decline [17], and up-regulate BDNF expression in the hippocampus following exogenous corticosterone treatment in rats [18]. Moreover; BRB could ameliorate dysfunctions of learning and memory with a neuroprotective effect in traumatic brain injury [19]. Mounting evidence focused on the dose-dependent neuroprotective effect of BRB, which can reduce depression symptoms by increasing serotonin and dopamine levels at a low dose [20].

Mahmoudvand et al. reported the potential prophylactic effect of methanolic extract of *B. vulgaris* root against murine acute toxoplasmosis induced by RH strain of *T. gondii* [14]. On the other hand, infections with *T. gondii* have been associated with the early onset of schizophrenia and BRB analogs have shown an inhibitory effect against *T. gondii* infection [12]. However, *T. gondii* alternations in BDNF expression in corticolimbic structures, in particular, whether BRB can improve its induced memory impairment have been less understood.

IA is associated with the integrated activity of the various parts of the brain, especially the hippocampus and mPFC [21]. Because of the association between chronic infection of *T. gondii* and cognitive disturbances, the critical role of the PFC and hippocampus in learning and memory [22], and considering the BRB protective effect against memory impairment, this study was undertaken to investigate whether BRB could ameliorate *T.gondii*- induced learning and memory deficit and also a Ket-induced rat model of schizophrenia in a passive avoidance task in the rat.

## Methods

### Animals and parasite

This experimental study was performed on 48 adults male Wistar rats weighing 200–230 g for setting up an animal model and 20 BALB/c mice 20–25 g for the preservation of the parasite. The animals were obtained from the Central Animal House, College of Pharmacy, University of Urmia, Northwest of Iran. Before the beginning of the experiments, animals were housed in standard plastic cages for one week for habituation and were kept in a controlled environment at room temperature (25 ± 10 °C) with a 12:12 h light/dark cycle. The rats were fed with standard rodent pellet food and water were provided ad libitum throughout the experimental period. All efforts were made to minimize the suffering of the animals in accordance with Ethics Committee recommendations.

The RH strain of *T. gondii* belonging to genotype type I was used to establish chronic toxoplasmosis in rats [23]. The parasite was obtained from the Department of Parasitology, Faculty of Medical Sciences, Tarbiat Modares University, Tehran, Iran. Tachyzoites (1 × 10^5^) of the parasite were inoculated in BALB/c mice by i.p passage and were collected 3–5 days post-inoculation. The harvested tachyzoites were mixed with PBS, filtered to remove peritoneal cell contamination, counted under microscopy with a 40× objective using a hemocytometer, and adjusted to 10^6^-10^7^/ml in saline [15, 16].

### Chemicals

Ket (10%) and BRB (Berberine chloride) were purchased from Alfasan, Poland, and Sigma Aldrich, USA company, respectively. In addition, Cyclosporin A (Sigma, Cat No. 3-13-59865) (Cyc-A) and Hydrocortisone acetate (HCA) (Aburaihan, Iran) were prepared commercially.

### Experimental groups

The rats were randomly divided into two main groups: infected (infected with *T. gondii*) and non-infected (experience any infection, treated with saline or Ket i.p for 5 days). Both groups were supplemented with BRB 25 mg/kg for 5 days by oral gavage after two months. BRB was administrated after the IA training session for evaluating its effects on IA memory consolidation. Each main group was divided into subgroups including (TOXO-SAL, TOXO-BRB, Cont-SAL, BRB, Ket-SAL, and Ket-BRB (n = 8 in each subgroup).

The infected group was i.p inoculated with each 0.5 ml PBS containing 1 × 10^7^ tachyzoites [24]. To enhance the infectivity, we immunosuppressed the rats with Cyc-A (40 mg/kg/d) and HCA (0.5 mg/kg/d) via i.p injection for 5 consecutive days. On the fifth day, the animals were inoculated with adjusted tachyzoites [25, 26].

Because Cyc-A-induced toxicity on the central nervous system occurs after long-term administration [27], we only used it for five days to support the reduction of side effects of Cyc-A in this study. In addition, Cyc-A was administered with HCA to normalize the side effects of Cyc-A [28]. In our previous pilot study (unpublished), we did not observe a significant difference in memory performance after five days between the control group and the rats that received Cyc-A and HCA.

### Direct microscopy counting of tachyzoites or tissue cysts and molecular test

Two-month post-inoculation, following the behavioral IA test, the rats were examined for tachyzoites and tissue cysts by impression smear, and polymerase chain reaction (PCR) of homogenized brain samples after euthanasia (as described below) of animals [29]. Two drops of brain homogenate were placed on slides and the number of tachyzoites or tissue cysts was counted under light microscopy [30].

DNA was extracted from 30 mg digested brain tissue by using DNA Extraction Kit (DNP™, Cat. no. EX6071, Tehran, Iran) according to the manufacturer’s instructions. PCR was performed using the 529 bp repetitive element gene as described previously [31]. Summarily, 10 µL master mix (PCR Mastermix, Cat. no. MM2062, TC Clone, Tehran, Iran), 2 µL of each TOX forward and reverse primers [TOXF (5ʹ–CGCTGC AGGGAGGAAGACGAAAGTTG-3ʹ) and TOXR (5ʹ-CGCTGCAGACACAGTGCATCTGGATT-3ʹ )], 5 µL extracted DNA, and 6 µL distilled water were mixed to consist of 25 µL reaction mixture and was amplified according to setup program in Thermal Cycler (Veriti™ Dx, Cat. no. 4,388,444, USA) as follows: first and final denaturation include 7 min at 94 °C and 30 s at 94 °C for 35 cycles, respectively, annealing for 30 s at 56 °C, and first and final extension at 72 °C for 30 s and 10 min, respectively. The standard RH strain of *T. gondii*, and distilled water (D.W) were used as positive and negative controls, respectively.

### Ket exposure as an animal model of schizophrenia

**Fig. 1:**
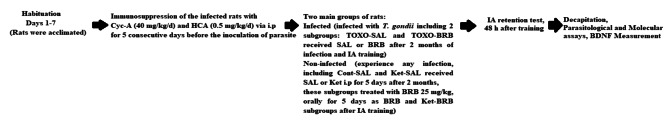
Time-line of the protocol used in all experiments, **Cyc-A**: Cyclosporin A; **HCA**: Hydrocortisone acetate; **TOXO**: toxoplsma; **SAL**: saline; **Cont**: control; **Ket**: Ketamine; **BRB**: Berberine; **IA**: inhibitory avoidance; **BDNF**: brain-derived neurotrophic factor

Ket (100 mg/mL) was diluted in saline to 30 mg/mL and injected i.p at a volume of 100 µl/kg of body weight. Rats were injected i.p once daily (late morning) with Ket for 5 consecutive days to induce a schizophrenia model in rats as a positive control [32]. Animals of the control group received saline in the same volume. Signs of ket exposure were observed after ket injection including decreased motivation. Also, learning impairment was observed in the IA test because of entering rats into a dark compartment after foot shock. In other words, when rats received ket for 5 days, and were then trained in an IA box, entrance into a dark area (decrease STL) occurred following placing each animal in a light compartment after shock delivery. Following Ket exposure, the rats were returned to the home cage and left undisturbed for drug washout before IA behavioral test for at least five days (see Fig. 1 for more details).

### Pavlovian fear conditioning

By using Pavlovian fear conditioning, learning, and memory were evaluated as previously described [33]. Animals were placed in a chamber and learned to associate a conditioned stimulus (CS) with an unconditioned stimulus (UCS). A passive avoidance test was carried out in the shuttle box apparatus (Tajhiz Ghostare Omid Iranian Company, IRAN) composed of two equal-sized light and dark compartments. Each chamber (32 cm wide × 25 cm high × 25 cm deep) had Plexiglas side walls, and a floor consisting of stainless-steel rods equipped to deliver foot shocks at 0.5 mA. A shock to the animal’s foot was delivered through the steel rods on the floor. Each conditioning compartment was surrounded by a sound-attenuating environmental chamber and equipped with an overhead camera to record the rat movements during each test. Both dark and illuminated chambers were gently cleaned with 70% ethanol between each animal test. Each rat was placed in the light compartment, allowing it to enter the dark area for habituating to the apparatus. On the training day, animals were conditioned in the light chamber allowed to pass the door, and followed by the 1s foot shock of 0.5 mA delivered shock via the floor grid. After the training session, the animals were returned to their home cage. 48 h later, via placing the rat in the illuminated same chamber, the guillotine door was opened 9 s later. Following the entrance of the animals into the dark box, the latency of entering into the dark compartment (Step-Through Latency, STL), and the time spent in the dark chamber for 10 min were recorded. 600 s as a maximum cut-off time was recorded in the case of entering into the dark compartment occurred [33].

### BDNF measurements

The rats were deeply anesthetized with carbon dioxide and rapidly sacrificed [34], then hippocampi and PFC of the brains were immediately collected and frozen at − 80 °C until used for BDNF measurements. The tissues were homogenized in cold lysis buffer and centrifugation at 12,000 g for 20 min at 4 °C [35]. The BDNF protein levels were assessed using Rat BDNF ELISA kits (PicoKine™, Cat. No. EK0308, USA) according to the manufacturer’s recommendations.

### Data analysis

All data were analyzed using the software SPSS (Version 26). The data obtained from the behavioral test were analyzed using a two-way analysis of variance (ANOVA), with two independent factors including infection (infected and non-infected) and treatment (vehicle and BRB). In the case of identified differences, the Tukey *post hoc* test was performed to describe specific differences between all groups. P < 0.05 was considered statistically significant.

## Results

### Direct microscopy counting of tachyzoites or tissue cysts and molecular test

**Fig. 2:**
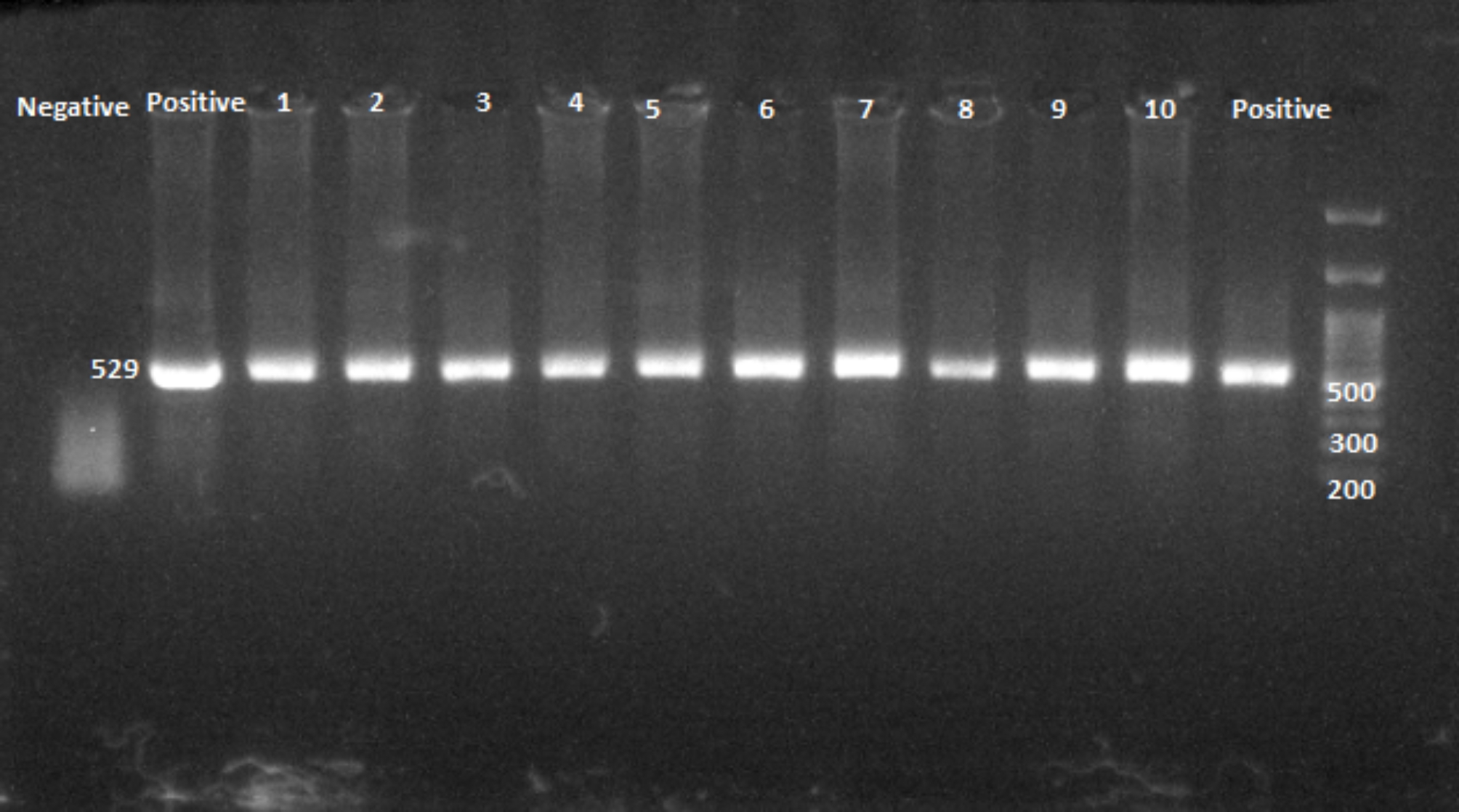
*T. gondii* DNA was detected by the using 529-bp repeat gene in rat brain tissues of chronic toxoplasma after two-month infection. Negative: D.W, Positive: RH strain of *T. gondii* DNA, 1–10: brain tissue DNA samples

We were not able to observe tachyzoites or tissue cysts by impression smear of brain tissue, but by using the 529-bp repeat gene, one of the most sensitive genes in the detection of *T. gondii*, it was detected in all infected rats that entered experiments (Fig. 2).

Additionally, in our previous pilot study (unpublished), we detected parasite DNA in brain homogenate during the experimental period.

### Ket exposure and ***T. gondii*** infection impaired long-term IA memory consolidation were improved by BRB administration

**Fig. 3:**
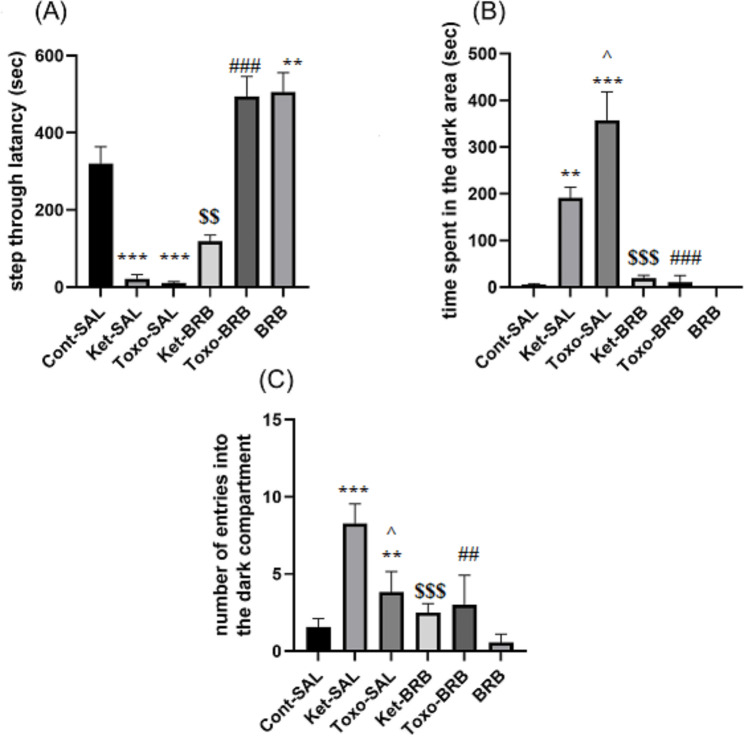
Effects of exposure to Ket and *T.gondii* infection on inhibitory avoidance consolidation in infected and non-infected rats. **(A)** STL, **(B)** time spent in the dark compartment, **(C)** and the number of entries to the dark compartment, (n = 8 rats in each group). **P = 0.01, ***P = 0. 001 compared to the control group, ^#^ P < 0.05, ^##^ P = 0.01, compared to Toxo-infected rats, ^$$^ P = 0.001 compared to the Ket-induced rats. **Cont**: control; **Ket**: Ketamine; **BRB**: Berberine; **STL**: Step through latency

To determine whether the BRB used at the dose of 25 mg/kg enhances IA memory consolidation in treated rats, we examined the IA memory performance 48 h after conditioning. The IA memory data of the experimental groups are illustrated in Fig. 3A two-way ANOVA for the STL showed a main effect of group (*F*_*2, 41*_ = 112.324, P = 0.001), main effect of treatment (*F*_*2, 41*_ =184.051, P = 0.001) and interaction between group and treatment (*F*_*2, 41*_ =39.071, P = 0.001). The post hoc analysis revealed that animals that were inoculated parasite or received Ket, showed lower STL compared to the control ones (P = 0.001), and exposure with BRB significantly increased STL in these groups (both, P = 0.001). As Fig. 3A illustrates, *T. gondii* infection or Ket impaired IA memory consolidation and decreased STL which could recover via BRB (P = 0.001) (Fig. 3A).

A two-way ANOVA for the number of entries into the dark area showed a main effect of group (*F*_*2, 42*_ = 18.748, P = 0.001), main effect of treatment (*F*_*2, 41*_ =22.347, P = 0.001) and interaction between group and treatment (*F*_*2, 41*_ =8.729, P = 0.001). As shown in Fig. 3B, Post-hoc comparisons showed that the number of entries into the dark compartment in the Ket-SAL group was significantly higher than in the SAL-SAL group (P = 0.001), demonstrating higher levels of memory deficit in the Ket-induced rats. Moreover, a significant difference was observed between the Ket-SAL and the Ket-BRB groups (P = 0.001) (Fig. 3B). Similarly, *T. gondii-* infected rats showed a higher number of entries into the dark Ket-induced group (P = 0.05).

A two-way ANOVA for the total time spent in the dark compartment revealed a significant effect of group (*F*_*2, 42*_ =26.231, P = 0.001), main effect of treatment (*F*_*2, 42*_ =71.552, P = 0.001) and interaction between group and treatment (*F*_*2, 41*_ =23.637, P = 0.001). As observed, all infected rats spent more time in the dark chamber compared to the control group (P = 0.001). Also, as shown in Fig. 3C, our results indicated increased time spent in the dark compartment in the animals that were injected with the parasite or received Ket compared to the control group (P = 0.001). Additionally, BRB decreased the time spent in dark areas in infected rats compare to infected ones (P = 0.001) indicating the potent property of BRB against schizophrenia-like cognitive deficits. *T. gondii*-infected rats spent more time in the dark area than the Ket-induced group (P = 0.05) (Fig. 3C).

### Ket exposure and ***T. gondii*** infection impaired long-term IA memory reconsolidation were improved by the BRB administration

**Fig. 4:**
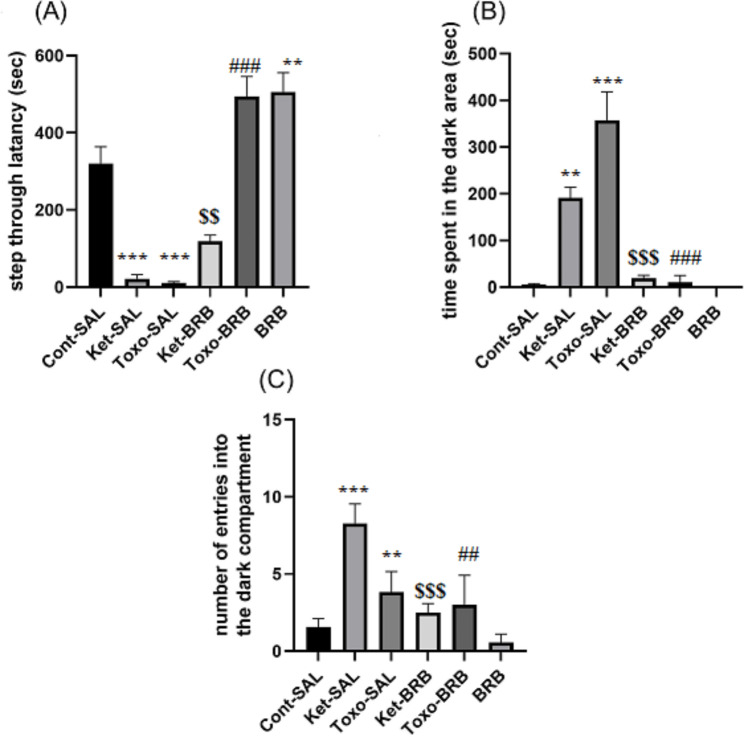
Effects of exposure to Ket and *T. gondii* infection on inhibitory avoidance reconsolidation in infected and non-infected rats. **(A)** STL, **(B)** time spent in the dark compartment, **(C)** and the number of entries to the dark compartment (Fig. 2A–C respectively) (n = 8 rats in each group). * P < 0.05, **P = 0.01, ***P = 0. 001 compared to the control group, ^##^ P = 0.01, ^###^ P = 0.001, compared to toxo infected rats, $$ P = 0.001 compared to the Ket-induced rats. **Cont**: control; **Ket**: Ketamine; **BRB**: Berberine; **STL**: Step through latency

In this experiment for evaluating determine whether the injection of BRB at the dose of 25 mg/kg enhances IA memory reconsolidation in treated rats, we examined the IA memory performance, 48 h after injection of the BRB. The reconsolidation of IA memory was impaired by the Ket exposure and *T. gondii* infection after memory retrieval/reactivation (Fig. 4). The IA memory data of the experimental groups are illustrated in Fig. 4A two-way analysis of variance (ANOVA) for the STL showed a main effect of group (*F*_*2, 42*_ =76.751, P = 0.001), main effect of treatment (*F*_*2, 421*_ =63.064, P = 0.001) and interaction between group and treatment (*F*_*2, 42*_ =20.010, P = 0.001). As illustrated in Fig. 4A, while Ket-Sal and Toxo-Sal groups had lower STL (P = 0.001), STL elevated in BRB and Toxo-BRB and Ket-BRB groups (P = 0.01, P = 0.001) compare to Cont group. Also, STL has significantly elevated in BRB-treated rats compared to controls (P = 0.01, P = 0.001). Therefore, the overall pattern of results demonstrates that *T. gondii* could impaire retrieval and subsequent consolidation of IA memory which could recover via BRB.

A two-way ANOVA for the total time spent in the dark compartment showed a main effect of group (*F*_*2, 42*_ =5.150, P = 0.01), main effect of treatment (*F*_*1, 42*_ =50.377, P = 0.001) and interaction between group and treatment (*F*_*2, 42*_ = 25.987, P = 0.001). Ket-Sal and Toxo-Sal groups spent more time in the dark compartment (P < 0.05). As shown in Fig. 4B, Post-hoc comparisons showed that BRB significantly decreased the total time spent in a dark area in rats infected with *T. gondii* (P = 0.001) and Ket-induced groups (P = 0.01) indicating Ket-induced memory impairment recovered by using BRB. There was a significant difference between BRB and control rats (P = 0.001). (Fig. 4B).

A two-way ANOVA for the number of entries into the dark area revealed a significant effect of group main effect of group (*F*_*2, 42*_ = 37.421, P = 0.001), main effect interaction between group and treatment (*F*_*2, 41*_ =39.389, P = 0. 001) but no effect of treatment (*F*_*2, 42*_ =1.004, P = 0.001). As observed, all rats injected with Ket or infected by *T. gondii* showed a high number of entries into the dark area compare to the control group (P = 0.001). Furthermore, we observed that BRB significantly decreased the number of entries into the dark area (P = 0.01). Moreover, no significant difference was observed between BRB and the control group (Fig. 4C).

### Ket exposure and ***T. gondii*** infection altered prefrontal cortex and hippocampal BDNF expression ***mPFC BDNF***

**Fig. 5:**
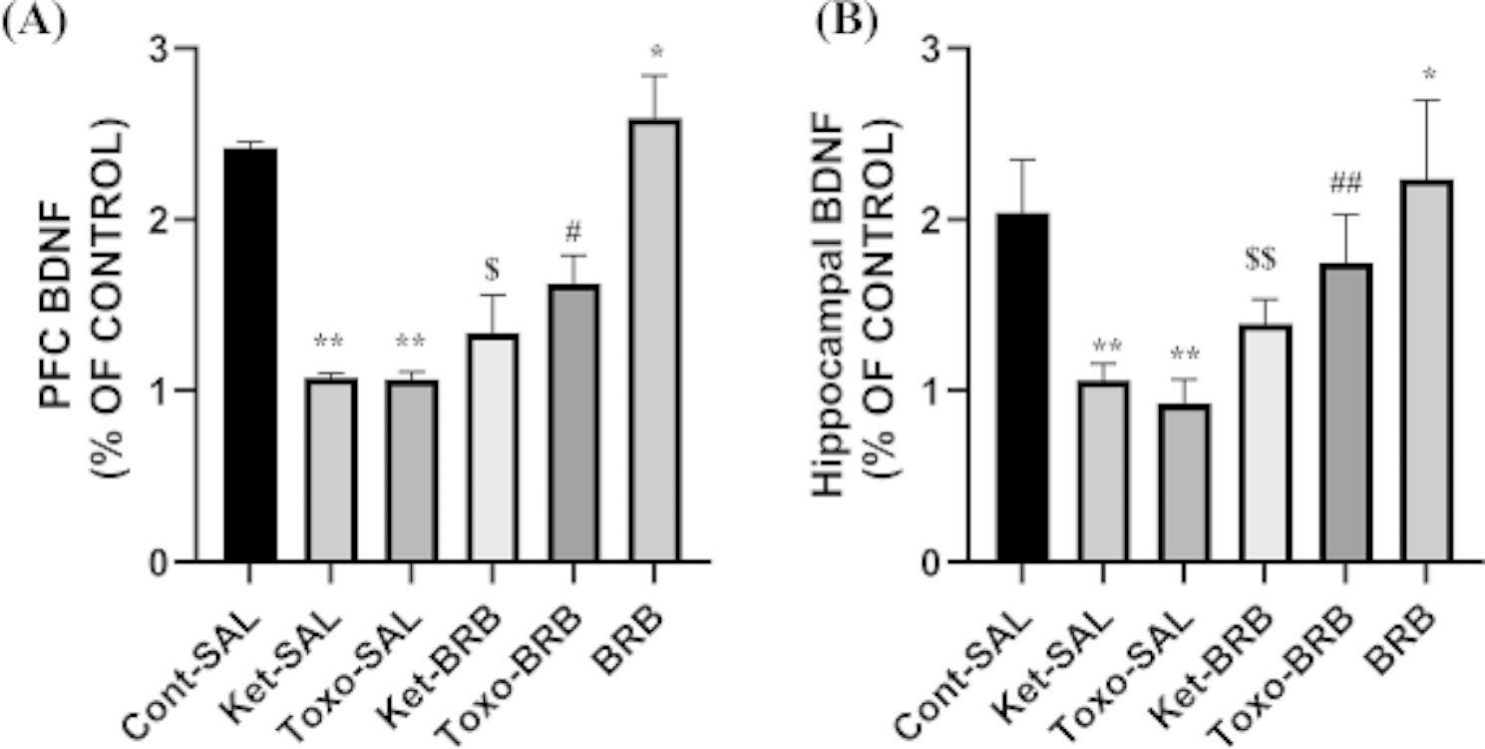
Effects of exposure to Ket and *T. gondii* infection on **(A)** BDNF expression in PFC and **(B)** in the hippocampus. * P < 0.05 and **P = 0.01 compared to the control group, ^#^ P < 0.05, ^##^ P = 0.01, compared to toxo infected rats, ^$^ P < 0.05 and ^$$^ P = 0.001 compared to the Ket-induced rats. **Cont**: control; **Ket**: Ketamine; **BRB**: Berberine; **STL**: Step through latency; **Cont**: control; **Ket**: Ketamine; **BRB**: Berberine; **BDNF**: Brain-derived neurotrophic factor; **PFC**: Prefrontal cortex; **STL**: Step through latency

A one-way ANOVA for mPFC BDNF (Fig. 5A) showed a significant effect of group (F _5, 29_ =94.421, P = 0.001). Between-group comparisons showed that the BDNF level in the PFC significantly decreased in all groups except rats that received BRB from that in the Cont-SAL group (P = 0.00, and P < 0.05). BDNF levels were significantly increased in the Toxo-BRB and Ket-BRB groups from those in the Toxo-SAL and Ket-SAL groups (P = 0.01), indicating protective effects of BRB on memory reconsolidation induced by parasite infection and Ket (Fig. 5A).

### Hippocampal BDNF

A one-way ANOVA for hippocampus BDNF (Fig. 5B) showed a significant effect of group (F_5, 29_ =13.836, P = 0.001). Between-group comparisons indicate that BRB increased BDNF levels in the hippocampus (P < 0.05). Moreover, between-group comparisons indicated that the hippocampal BDNF significantly decreased in the Ket-SAL and Toxo-SAL groups from that in the Cont-SAL group (both, P = 0.001). BDNF levels were significantly increased in the Toxo-BRB and Ket-BRB groups from those in the Toxo-SAL and Ket-SAL groups (P = 0.001) (Fig. 5B).

## Discussion

To our knowledge, the current work is the first study to investigate the *T. gondii* infection in a schizophrenia-like rat model to examine its effect on IA memory consolidation and reconsolidation. To consider toxoplasma as one of the probable causes of schizophrenia, some seroepidemiological studies displayed high serum anti-toxoplasma antibodies in these patients [36, 37]. However, studies showed the relationship between toxoplasmosis and brain damage in an animal model of schizophrenia by behavioral tests [38]. But there is no information on the effect of BRB on the memory deficits induced by chronic *T. gondii* infection. So, we decided to study whether BRB supplementation will increase the memory deficits induced by chronic *T. gondii* infection and the Ket-induced animal model of schizophrenia. For this purpose, we made chronic toxoplasmosis in the rat, the best model for human chronic toxoplasmosis studies [23, 24]. Because rat is resistant to *T. gondii* RH strain as well as other brain murine parasites, we immunosuppressed the animals before inoculation the parasite. Some of the experimental studies showed the large size of brain cysts in rats using Cyc-A [39]. Highlighting the finding of Asgari et al., no tissue cyst had been found in impression smears of rat brains [35], but we detected the parasite DNA in the brain tissue homogenate.

Concerning the use of 30 mg/kg Ket induces some noticeable changes in behavior and elements involved in neurotransmissions related to schizophrenia, we used the same dose for an animal model of schizophrenia [32].

BDNF is considered a crucial neurotrophic factor and it has been proven to be a fundamental factor in synaptic plasticity and neurodevelopmental programming, playing an important role in memory consolidation [40, 41]. The experimental data obtained from this work are in line with previous evidence [42], supporting the role of the BDNF on memory processing and lowering the hippocampal and PFC BDNF expression in infected as well as Ket-treated rats. We showed that BRB at a memory-enhancing dose has a significant effect on BDNF expression in two memory-processing-dependent areas of the brain including mPFC and hippocampus. Elevations of BDNF in the brain circuits involved in IA memory consolidation and reconsolidation following BRB supplementation can supply a potential role in *T. gondii-*induced memory impairment therapies.

One potential pathophysiological mechanism underlying the progression of schizophrenia seems to depend on dysfunction in the N-methyl-d-aspartate glutamate receptor (NMDAR) [43]. The NMDA model of schizophrenia originated from the observation that NMDA antagonists, like Ket, can induce memory impairment. Many previous studies illustrated that chronic toxoplasma infection may be contributed to human behavior changes, obsessive-compulsive disorder, or even schizophrenia [5]. Cognitive disturbances are a major feature in patients with schizophrenia due to the infection-induced serious problems in brain functions [32]. Meanwhile, *T. gondii* infection is associated with human behavior and personality alternations and suicide so can be a risk factor for developing mental disorders such as schizophrenia and depression [2, 44]. Also, the infection can result in other neurodegenerative symptoms such as memory impairment [7] and cognitive deficits [8]. According to the obtained results from the IA memory test, *T. gondii* infection caused schizophrenia-like symptoms and has detrimental effects on IA memory consolidation. This finding provides potential strong evidence that such infection targeting PFC function may play a critical role in higher-level cognitive function in the context of schizophrenia. Consistent with our findings, Zhou et al. (2011) have indicated that *T. gondii* Prugniaud strain-induced chronic toxoplasmosis leads to disturbances in learning and memory performance [45]. In the other study performed by Daniels et al. (2015), latent toxoplasmosis associated with neurocognitive symptoms, especially remarkable memory deficit has also been reported in infected rats [46]. In addition, *T. gondii* can influence all brain regions, yet the cortex especially exhibits more severe tissue damage compared with the other areas [47]. Some studies indicate that cortico-limbic structures including the PFC and hippocampus are necessary for the encoding as well as early recall of associative memories including fear memories [47, 48].

Interestingly, BDNF critically contributed to memory consolidation in a number of learning paradigms [49]. In addition to its well-established contribution to neuronal proliferation, survival, and memory consolidation, BDNF mediates hippocampus-dependent reconsolidation memory formation [50, 51]. Here, we confirmed that reactivation in the shuttle box apparatus initiates reconsolidation in the hippocampus, and also illustrated that BDNF is involved in this process, controlling the BRB effect on deficits in memory performance induced by infection. We also showed evidence presenting that BRB improved IA memory consolidation through elevations in BDNF levels in the hippocampus and PFC areas, and that activation of BDNF signaling after IA reactivation reverses the memory disturbances caused by *T. gondii* infection. In addition to its effect on memory consolidation, *T. gondii* was also modestly effective in attenuating reconsolidation, providing an additional therapeutic avenue for treating negative symptoms of schizophrenia, perhaps through lowering the BDNF expression in fear-related brain areas including PFC and hippocampus. To address the effect of *T. gondii* infection on the memory reconsolidation impairment and protective effects of BRB, the present study further emphasizes the important role of the BDNF in IA reconsolidation and identified BRB as an important mediator for fear memory reconsolidation in the infected rats. It is therefore reasonable to hypothesize that *T. gondii* can affect NMDAR function and glutamatergic neuronal circuits.

Similarly, we demonstrated a positive correlation between BDNF levels in the hippocampus and mPFC as well as memory impairment in the Ket-induced model, as assessed by the IA test. The current study support that BDNF alternations may be closely associated with impaired memory and cognitive function. Another possible explanation for this result may depend on the influence of the Ket on NMDAR function in the brain. Multiple studies have reported that the N-methyl-D-aspartate receptors (NMDARs) in the hippocampus, as ionotropic glutamate receptors, contribute to memory formation [22, 52]. Furthermore; the NMDAR agonists promote and their antagonists decrease hippocampal-dependent memory consolidation and retrieval [52, 53]. In this study; we observed diminished memory performance in Ket-treated rats suggesting Ket, as an NMDA receptor antagonist should prevent the memory performance. As a consequence, BDNF level in the hippocampus is also reduced in the Ket-induced model and *T. gondii* infected rats supporting again the memory impairment in the IA task. These results suggest that BRB may be useful as a therapeutic agent for improving memory deficits by stimulating BDNF expression and alleviating memory performance.

## Conclusions

In conclusion, the findings of the current study indicated that chronic toxoplasmosis infection in an animal model has a detrimental effect on IA memory performance and reduces BDNF levels in PFC and hippocampus. Also, infection with *T. gondii* or Ket-induced schizophrenia rat model influenced by BRB, so that BRB significantly improved deficits in memory processing induced by infection. Our study suggests that BRB can alleviate memory deficits induced by *T. gondii* infection or Ket, probably through the alterations in BDNF expression that might be involved in the behavioral changes leading to schizophrenia. Therefore, BRB may act as a strong agent that could be clinically relevant in the treatment of schizophrenia.

## Data Availability

All data and material are available from the corresponding author to share upon request.
